# What Could Arrest an Eriophyoid Mite on a Plant? The Case of *Aculops allotrichus* from the Black Locust Tree

**DOI:** 10.3390/insects12111031

**Published:** 2021-11-16

**Authors:** Katarzyna Michalska, Marcin Studnicki

**Affiliations:** 1 Section of Applied Entomology, Department of Plant Protection, Institute of Horticulture Sciences, Warsaw University of Life Sciences, Nowoursynowska 159, 02-776 Warsaw, Poland; 2 Department of Biometry, Institute of Agriculture, Warsaw University of Life Sciences, Nowoursynowska 159, 02-776 Warsaw, Poland; marcin_studnicki@sggw.edu.pl

**Keywords:** predation risk, competition, alarm cues, arrestment, attraction, host plant recognition

## Abstract

**Simple Summary:**

For a phytophagous arthropod, plant cues and the presence of conspecifics or heterospecifics on a plant are important sources of information about food availability and predation risk. In eriophyoid mites, which are a group of economically important plant parasites, this issue is still poorly understood. In the previous study, females of the eriophyoid *Aculops allotrichus* prolonged their stay on unprofitable, old black locust leaves with injured conspecifics, suggesting their specific attraction to pierced individuals. The aim of this study was to shed light on this phenomenon. The series of tests using intact and artificially injured conspecifics, heterospecifics, pollen and sand grains revealed the high specificity of cues associated with mite injury. Only the presence of conspecifics triggered female arrestment on unprofitable old leaves, and this effect was more pronounced on leaf patches with injured than with intact eriophyoids. To our best knowledge, *A. allotrichus* is the first described herbivore in which injured conspecifics, instead of causing an alarm to be raised, keep the foraging individuals within a risky patch. Our tests using three non-host plants also suggest that *A. allotrichus* presumably needs more time to identify and evaluate non-host plants than host plants.

**Abstract:**

*Aculops allotrichus* is a vagrant eriophyoid that lives gregariously on the leaves of the black locust tree. This study demonstrated that conspecifics can have a significant impact on *A. allotrichus* females on unprofitable, old black locust leaves and can arrest them on those leaves. The effect was more pronounced in females that were exposed to artificially injured individuals than to intact ones. They not only prolonged their sojourn on leaf discs with pierced conspecifics, but also preferred the leaf disc halves with damaged individuals to clean ones. *Aculops allotrichus* is the first described herbivore in which artificially injured conspecifics, instead of causing alarm, keep the foraging individuals within a risky patch. Other objects, such as artificially injured or intact heterospecifics, pollen or sand, were irrelevant to the eriophyoid females on old leaf patches. In tests with old leaves of maple, magnolia and hard kiwi vine, the females postponed their movement from non-host leaf discs, which suggests that they may need more time to recognise and evaluate unfamiliar plants than familiar ones.

## 1. Introduction

For a herbivore arthropod, a host plant constitutes a food source and often also a place to live. The web of information it uses is complex and can be utilised or exploited by other members of the multitrophic system [1,2,3,4]. Plant cues (chemistry and morphology-borne) can provide information about the quality and quantity of food, shelter and also the presence of other herbivores through herbivore-induced plant volatiles [5,6,7]. The odours of conspecifics can also signal potential food resources, protection against enemies or the presence of mates [8,9]. Nonetheless, con- and heterospecifics often compete for plant resources, and their cues may elicit avoidance behaviour rather than attraction [10,11]. The avoidance or escape response can be also triggered by carnivore cues or injured conspecifics, raising the alarm to a herbivore about the presence of an enemy on plants [12,13,14]. All these cues appear to be important for foraging and host-plant selection by phytophagous arthropods [1]. 

Eriophyoid mites are tiny (0.2–0.4 mm long), mostly monophagous plant parasites that are vagrants, inhabit refugia on plants or induce galls within those which they live [15,16,17]. Several species are of great economic importance in transmitting plant viruses and severely injuring crop plants [17]. 

The role of infochemicals in shaping eriophyoid interactions with conspecifics, heterospecifics and plants is not yet fully understood [18,19,20,21,22]. Within a host plant, eriophyoids disperse by walking, while over longer distances, they are mainly dispersed passively by wind [16,19]. Their host plant creates a mosaic of more or less profitable sites that may additionally be occupied by conspecific or heterospecific competitors, such as other eriophyoid species, spider mites, tarsonemids, thrips and caterpillars, or predators, mainly phytoseiid mites [23]. Moreover, when dispersing aerially, the eriophyoids may land on a novel, non-host plant and, on the basis of its cues, they may decide whether to resume dispersion or not. Investigations on *Abacarus hystrix* (Nalepa) suggested that eriophyoids can quickly recognise non-host plants. When landing on a non-familiar plant, *A. hystrix* increased its mobility, running all over the plant and showing a tendency to disperse [24]. However, in further investigations by [21], only four out of ten of the tested eriophyoid species became more active on non-host plants. Recent observations on *Aculops allotrichus* (Nalepa) [25] and *Aceria guerreronis* Keifer [20] revealed that eriophyoids can also respond to the cues left on plants by predatory mites and heterospecific competitors. The eriophyoids of *A. guerreronis* sought refugia under floral bracts of the coconut when they were exposed to the cues of phytoseiids, *Neoseiulus baraki* (Athias–Henriot), or *Amblyseilus largoensis* (Muma) [20]. However, they remained outside the refugium in the presence of the cues of the competitor, the tarsonemid mite *Steneotarsonemus concavuscutum* Lofego and Gondim Jr. Males of *A. allotrichus* also responded to predation risk and decreased their spermatophore deposition rate in the presence of cues from the phytoseiid mite *Amblyseilus swirskii* (Athias–Henriot) [25]. Interestingly, the presence of artificially pierced conspecifics did not elicit either movement of *A. guerreronis* to the safer sites [20] or changes in the spermatophore deposition rate in *A. allotrichus* males [25]. In further tests, females of *A. allotrichus* also did not exhibit anti-predatory behaviours, such as freezing, escape or avoidance of oviposition on leaves with injured conspecifics [22]. On the contrary, the females that were exposed to the presence of damaged individuals on old leaves (not previously infested by eriophyoids) postponed their movement from those leaves [22]. Such female arrestment suggested the attraction of the eriophyoids to the odours emitted from damaged conspecifics and their orientated movement towards them [22]. This is in contrast with the spider mite, *Tetranychus urticae* Koch, in which females not only avoided leaf patches with phytoseiid cues, but also with artificially damaged conspecifics and diminished egg laying on such leaves [12].

Injured or killed individuals are an important source of information about the risk of predation for many invertebrates and vertebrates [12,26,27,28,29]. However, there are several examples of species that did not react to alarm cues alone or responded to them to a lesser extent and/or magnitude than, for example, to kairomones [30]. Moreover, for some cannibalistic species or scavengers, information about injured or dead conspecifics does not indicate predation risk, but rather the presence of food or shelter [31,32,33].

This study aimed at examining (1) the possible attraction that injured conspecifics hold for the herbivore mite *A. allotrichus*. We also tested the arrestment and/or attraction towards intact conspecifics, and the response of *A. allotrichus* to the cues of several other objects on leaves, including (2) injured or intact heterospecifics and (3) non-animal objects, such as sand and pollen grains. Finally, we examined (4) the effect of the cues of non-host plants, whose presence could evoke arrestment or escape in this mite. 

Re (1): *Aculops allotrichus* is a vagrant eriophyoid that lives gregariously on the compound leaves of the black locust tree, *Robinia pseudoacacia* L. (Fabales: Fabaceae). The eriophyid prefers very young, already unfolded leaflets of the black locust for settlement [34]. Nonetheless, it also forms dense populations on older leaves, though most probably as a consequence of its earlier invasion of young leaves [22]. We hypothesised that injured or dead individuals present on old and non-infested leaves could signal the presence of the eriophyoid colony and attract females to those leaves. As living individuals could also be a cue of a colony, apart from pierced individuals, we also tested the arrestment or attraction by intact eriophyoids on old leaves. 

Re (2): injured heterospecifics can be a source of alarm cues not only for closely related, but also for distant species, sympatric or allopatric [35,36,37]. In our study, we used heterospecific mites and insects, which had come from the black locust tree or from a non-host. As *A. allotrichus* did not exhibit anti-predatory behaviours in the presence of injured conspecifics [22], we hypothesised that the mite would not respond to injured heterospecifics either. However, we did not exclude the possibility that intact heterospecifics could be competitors for this mite, and that their presence would accelerate the movement of *A. allotrichus* females from the leaves. 

Re (3): we examined the movement of *A. allotrichus* females from black locust leaves in the presence of sand and pollen originating from two non-host plants. Under natural conditions, both these objects could have drifted with the wind onto a black locust leaf surface and got caught by leaf trichomes on a leaf blade. We expected that they could postpone the movement of females from leaves. However, while sand grains are mostly only physical obstacles for walking eriophyoids, pollen introduced a novel odour onto a leaf, which could additionally modulate the exploratory behaviour of the mite.

Re (4): when landing on a novel, unsuitable plant, an eriophyoid needs to continue dispersion in order to find its host. The act of further dispersion, however, does not guarantee that the eriophyoid will find a familiar plant, especially when the latter does not grow in the proximity [24]. While the cues of a familiar, but not preferred, old leaf should trigger the quick movement of eriophyoid mites away from that leaf to find a more suitable one within a host plant, on an unfamiliar plant leaf, the eriophyoids may need more time for non-host recognition. As a consequence, they may prolong their stay on such a leaf. We verified this hypothesis by testing *A. allotrichus* females on the leaves of three plant species, which are non-hosts for this eriophyoid. 

## 2. Materials and Methods

### 2.1. Study Objects and Plants

The experiments were conducted using *A. allotrichus* females that were collected from leaves of the black locust tree, *R. pseudoacacia,* grown on the campus of the Warsaw University of Life Sciences (WULS), Poland. The eriophyoids were tested on the leaves of the black locust and three unfamiliar, non-host plants: the hard kiwi vine, *Actinidia arguta* (Ericales: Actinidiaceae), the magnolia tree *Magnolia* × *soulangeana* Soul.-Bod. cv ‘Susan’ (Magnoliales: Magnoliaceae) and the maple tree, *Acer platanoides* L. (Sapindales: Sapindaceae). All these plant species belong to different plant orders and are phylogenetically distant from each other [38,39]. 

Apart from *A. allotrichus,* we also used the following categories of objects: (1) heterospecifics from the black locust and from a non-host plant, (2) pollen from non-host plants, and (3) sand. The heterospecifics were herbivorous insects and mites: the cowpea aphid, *Aphis carccivora* Koch and the two-spotted spider mite *T. urticae*, both from the black locust, *T. urticae* from the common bean (*Phasoleus vulgaris* L.) cv ‘Ferrari’, the vagrant eriophyoid mite *Cecidophyopsis hendersoni* (Keifer) from the spineless yucca *Yucca gigantea* Lem, and an omnivorous mite, the mould mite, *Tyrophagus putrescentiae* (Schrank), reared on yeast flakes. The cowpea aphids are polyphagous insects, which are widely distributed all over the world and also recorded on *R. pseudoacacia* in Poland [40]. In this study, the aphids were collected from the leaves of the black locust tree grown in the Ursynów district of Warsaw, Poland. They were put onto microscopic slides and identified, using keys to aphid species identification [41,42]. The stock population of the two-spotted spider mite originated from the mass rearings conducted on the bean plants cv ‘Ferrari’ in the Department of Plant Protection at WULS. The bean seed material was obtained from the commercial seed company, PNOS Ożarów Mazowiecki (Poland). The ‘bean’ population of spider mites was maintained on the potted bean plants kept in a growth chamber, while the ‘black locust’ population was maintained on the potted seedlings of the black locust. *Robinia pseudoacacia* is widespread in Poland [43] and it is neither under strict or partial protection [44], nor on the list of alien plant species capable of threatening native species or natural habitats [45]. To obtain the ‘black locust’ spider mites in spring, we collected the black locust seedlings from the field and transferred them to the glasshouses at WULS. Then, the plants were potted and infested by the ‘bean’ population of spider mites, which developed on leaves of the black locust for the following two months. The mould mite came from a mite colony reared on yeast in the Department of Plant Protection (WULS), and *C. hendersoni* came from the leaves of spineless yucca plants. The yucca plants were obtained from the commercial nursery of the ornamental plants, HRS Dawidy, Poland, and had been grown in glasshouses at WULS since 2000. The identification of *C. hendersoni* was made by Prof. Jan Boczek (Department of Plant Protection, WULS). In the present study, the taxonomic status of the eriophyoid was confirmed on microscopic slides using [46]. 

In the tests, we also used sand, which was collected from the Vistula River banks in Warsaw, and pollen originated from the two non-host plants, i.e., the cattail *Typha* sp. and the rape, *Brassica napus* L. subsp. *napus*. The cattail pollen was sampled from plants growing in Moczydło Park in Warsaw (Poland), while the rape pollen was obtained from the bee yard ‘Miody Bartkowiaka’ (Konarzewo, Poland). As the rape pollen was harvested commercially as pollen loads, before its use in the experiments, it was additionally ground using an electric coffee mill. The pollen was then stored at −30 °C. 

The leaves of the black locust, the magnolia, the maple tree and the hard kiwi vine were all obtained from plants that grew on the campus of WULS. The hard kiwi vine leaves were sampled from the plants grown in the Actinidia collection of the experimental orchard of the Department of Plant Protection and Dendrology (WULS) and identified by Dr. Piotr Latocha (Department of Plant Protection and Dendrology, WULS). The magnolia leaves were obtained from the magnolia collection maintained by the Department of Ornamental Plants (WULS) and identified by Dr. Jan Tonecki (Department of Ornamental Plants, WULS). The black locust and maple leaves were identified using [47]. The leaves of all plants were non-infested by insects, mites or pathogens and were relatively old (fully expanded, tough and deep green). They were picked from non-growing shoots of the plant and transported to the laboratory, using a portable refrigerator at 10 °C. The voucher specimens of insects and mites as well as the plants used in this study were deposited at the Section of Applied Entomology, Department of Plant Protection, WULS. 

### 2.2. General Methods

The experiments were conducted using single *A. allotrichus* females randomly selected from a population. Similar to our previous study [22], the females were tested on leaf discs of 6 mm in diameter that were cut off the ‘clean’ leaves, using a cork borer. Both the con- and heterospecifics were pierced with a fine, tip-sharpened needle, which usually led to the partial outflow of their body content and prompt death. To avoid damage to the leaf tissue during piercing, insects and mites were injured on a glass and then put onto the experimental leaf discs. The whole process, from piercing to the placement of the injured individuals on a leaf disc usually did not exceed 10–15 min, which ensured the relative ‘freshness’ of the killed insects and mites that were used in the test. All objects were transferred to the discs with a fine brush, except the eriophyoids, which, due to their microscopic size, were transported by means of an eyelash glued to a wooden stick. The observations were carried out on the abaxial side of a leaf, under a stereo microscope fitted to a cool light source. The time of female sojourn on a leaf disc was measured, using a stopwatch. 

**Figure 1:**
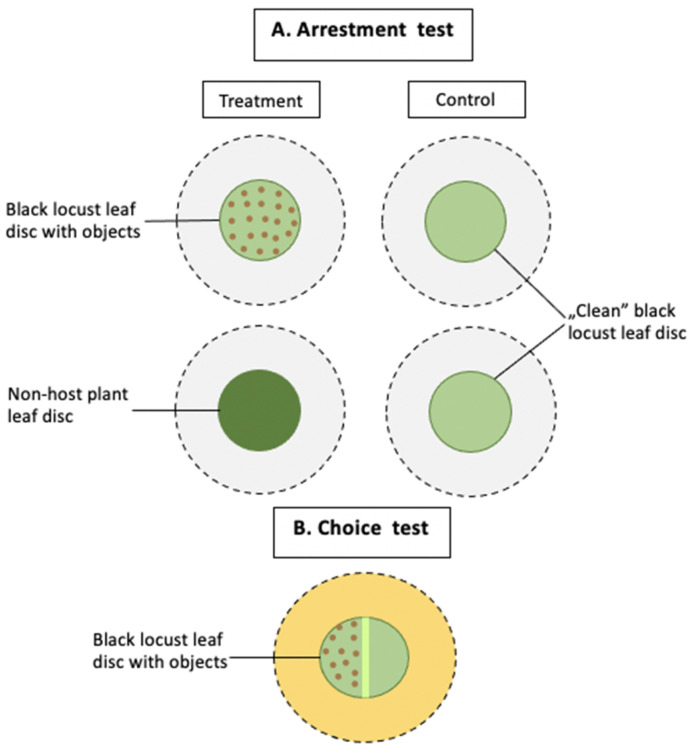
Schematic diagram of the experimental setup. (**A**) In the arrestment test, a female of *A. allotrichus* was released onto a leaf disc placed on the glass (pictured in grey). The treatment situation was either a black locust leaf disc, which contained objects (intact conspecifics, pierced or intact heterospecifics, pollen or sand) or a non-host plant leaf disc. The control was a ‘clean’ black locust leaf disc. (**B**) In the choice tests, a black locust leaf disc with the main vein running down the middle was placed onto a mat soaked with water (pictured in yellow). A female of *A. allotrichus*, released onto the middle of the vein, chose between one half of the disc, containing objects (pierced or intact conspecifics), and the second half, which was clean.

We carried out two types of experiments: the arrestment and two-choice test (Figure 1). As in the previous study [22], we indicated the arrestment of single *A. allotrichus* females by the presence of injured conspecifics on old black locust leaves; in the present tests, we examined whether intact conspecifics, injured or intact heterospecifics, sand or pollen grains could also detain eriophyoid females on such leaves. Additionally, we tested whether a female placed on the leaf patch of a non-host plant would accelerate or rather delay her movement from the ‘unfamiliar’ leaf patch. In the two-choice tests, we examined whether the *A. allotrichus* female could be attracted to the presence of conspecifics on old black locust leaves, and whether when it has a choice between the patch with or without conspecifics (injured or intact), it will choose the patch with the conspecifics. All tests were carried out in a climatic room at 26 ± 1 °C and 60–80% RH.

### 2.3. Arrestment by Objects on Black Locust Leaves and by Non-Host Leaves 

**Figure 2:**
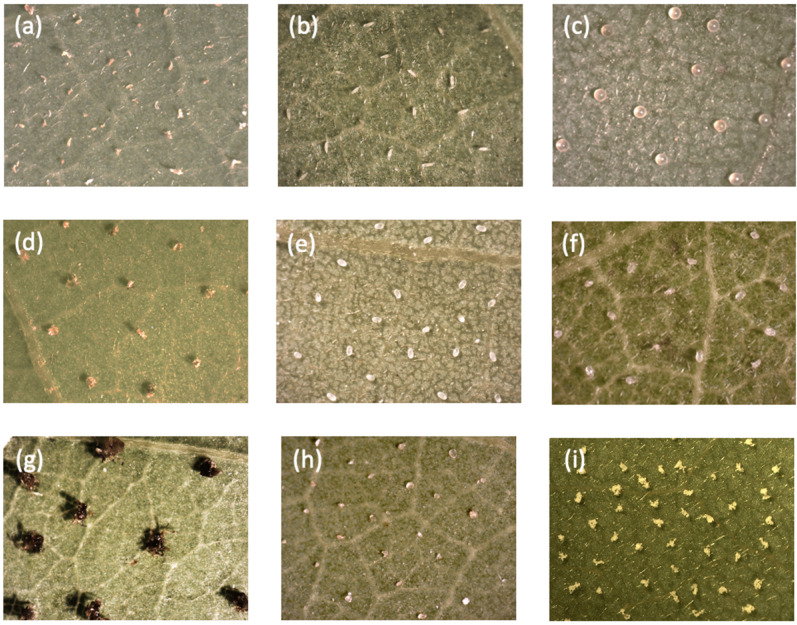
Microphotographs of various objects placed on black locust leaf discs in the tests: (**a**) pierced adults of *A. allotrichus*, (**b**) intact quiescent nymphs of *A. allotrichus*, (**c**) eggs of the two-spotted spider mite, (**d**) pierced larvae of the two-spotted spider mite, (**e**) eggs of the mould mite, (**f**) pierced larvae of the mould mite, (**g**) injured larvae of the cowpea aphid, (**h**) sand grains, and (**i**) aggregations of cattail pollen.

In these tests, we compared the time after which a single female of *A. allotrichus* abandoned the old black locust leaf discs with the particular objects, or non-host leaf discs, with the time in the control situation (‘clean’, non-injured leaf discs of black locust, without objects) (Figure 1). The non-host plants were hard kiwi vine, maple or magnolia, while the objects were intact *A. allotrichus*, intact or injured mould mites, intact or injured spider mites, injured eriophyoids of *C. hendersioni*, injured aphids, rape and cattail pollen and ‘odourless’ sand grains (Figure 2). The heterospecifics came from the black locust (aphids and spider mites) or from non-host (spider mites, mould mites and the eriophyoid of *C. hendersoni*). 

The experimental set-up was similar to that described by [22]. Leaf discs were cut off the leaf blade, avoiding the main veins. In the tests using objects, we took two leaf discs from each black locust leaflet. They were cut off a leaf blade on the opposite sides of a main vein. One disc was used for the control and the second one with the particular kinds of objects for a treatment combination. By contrast, in the ‘non-host plant’ test, the leaf disc cut from the old non-host leaf was a treatment combination, while that from the old black locust leaflet was a control. 

After being cut off, each leaf disc was placed centrally onto the glass of the upper side of a Petri dish lid (6 cm in diameter). The bottom of discs (the upper side of a leaf) was previously moistened with water to enable better attachment of the leaf surface to the glass and to limit leaf desiccation. As soon as the discs were attached to the glass and the objects were transferred and evenly distributed on the discs, the single females were released onto them. We regarded a female as abandoning a leaf disc if she entered a glass with all four legs.

In the previous experiments [22], we placed ca. 80 pierced conspecifics (mostly adults of *A. allotrichus*) per disc, which simulated a relatively high risk of predation. To make the present tests comparable with the previous experiments, we adjusted the size and/or number of the objects to the size of the injured eriophyoids and the space they occupied on a leaf disc (Figure 2). Very small objects, such as pollen grains, were put in aggregations of over a dozen grains, and ca. 80 such aggregations were placed down per disc. The sand grains chosen for the test (after sieving the sand sample through 0.2–0.05 mm mesh) were of a similar size to pierced eriophyoids (0.1–0.2 mm long) and were also used in a similar density to that of injured eriophyoids. Other objects, which were bigger than the eriophyoids, were chosen at the youngest stages (eggs or larva) and applied in a lower density. In the experiment with the injured heterospecifics, we used the 1st instar larvae of cowpea aphids, larvae of spider mites, larvae of mould mites and the adults of the eriophyoid *C. hendersoni*. In the tests with the intact conspecifics and heterospecifics, the immobile mite stages were applied, i.e., quiescent nymphs of *A. allotrichus,* the eggs of spider mites and the eggs of mould mites.

In our experiments, the sand grains were ‘odourless’ and constituted only physical obstacles for the tested females. To remove any organic contamination, they were roasted at 300 °C before the tests. Other objects, apart from being a possible hindrance for walking females and the source of novel cues (e.g., the pollen of non-host plants) might have signalled the presence of an eriophyoid colony (intact quiescent nymphs of *A. allotrichus*), danger (injured heterospecifics) and the presence of competitors for food and space (intact heterospecifics). For each combination, we carried out *n* = 15 replications, except for the test with the intact *A. allotrichus,* which was replicated *n* = 16 times, and the tests with the injured *C. hendersoni* and the spider mites from the black locust, which were replicated *n* = 18 each.

### 2.4. Attraction of Intact and Injured Conspecifics. The Choice Tests

In these tests, single leaf discs were cut off the black locust leaflets in such a way that the main vein of a leaflet was situated in the middle of each leaf disc and divided the disc into two equal leaf halves (Figure 1). The discs were then placed in the centre of a Petri dish 6 cm in diameter on a mat (Vileda^®^, 100% natural, cellulose and cotton cloth) soaked with water. A shallow layer of water fringed the edges of the disc, limiting leaf dessication and female movement from a disc. To determine whether eriophyoid females would be attracted to injured or intact conspecifics, on one half of the disc, we put either (1) 40 injured adults or (2) 40 intact quiescent nymphs of *A. allotrichus,* while the second half of the disc was clean. Soon after the preparation of the disc with the particular combination of conspecifics, we released single females into the middle of the main vein and observed their walking continuously until they descended the vein and chose one of the two halves of the disc. This constituted the first choice of the females. Further female choices were determined on the basis of consecutive examinations of the female presence on each disc half, performed at one-minute intervals throughout the subsequent 30 min from the moment of the female’s first choice. We carried out *n* = 18 replications of the choice test with intact conspecifics and *n* = 19 replications with the injured conspecifics of *A. allotrichus.*

### 2.5. Statistical Analyses

In the non-choice tests, the effect of the presence of conspecifics, heterospecifics, pollen and sand grains or non-host plants on the time spent by females on the leaf disc was analysed, applying the generalised linear model (GLM) with gamma error distribution and log link function. In the choice tests, we assumed the first female choice between the treatment disc half (with injured or intact conspecifics) and control (clean) leaf disc half to be random and analysed it, using a two-sided binomial test. To estimate the general site preference of single females choosing between treatment and control leaf disc halves, the data recorded for each mite were pooled, and the Wilcoxon signed-rank test was applied. All statistical analyses were performed, using R 4.4.1 software [48]. The data are given as mean ± SE.

## 3. Results

During the experiments, females walked freely among all types of objects, which were placed on a black locust leaf disc. Some females also examined the objects, usually only a few of them, during their sojourn on a leaf disc by touching or scratching them briefly with one leg. On leaf discs with cattail or rape pollen, the females occasionally walked through the aggregations of grains. When some pollen grains accidently became attached to their legs, the eriophyoids slowed their movement down and, waving their legs vigorously, removed the grains while walking. 

In the arrestment test, *A. allotrichus* females spent a significantly longer time with intact conspecifics on the old black locust leaf discs than on the clean (control) leaf discs (Table 1). By contrast, the presence of other objects i.e., injured *C. hendersoni* from yucca, intact or injured spider mites either from black locust or from bean, injured or intact mould mites from yeast, injured aphids from black locust, rape and cattail pollen, or sand, had no significant effect on the time of the females’ sojourn on the old black locust leaf discs (Table 1). 

As shown in Table 2, the females stayed for significantly longer periods on the old, hard kiwi vine, magnolia or maple leaf discs than on the old black locust leaf discs.

**Figure 3:**
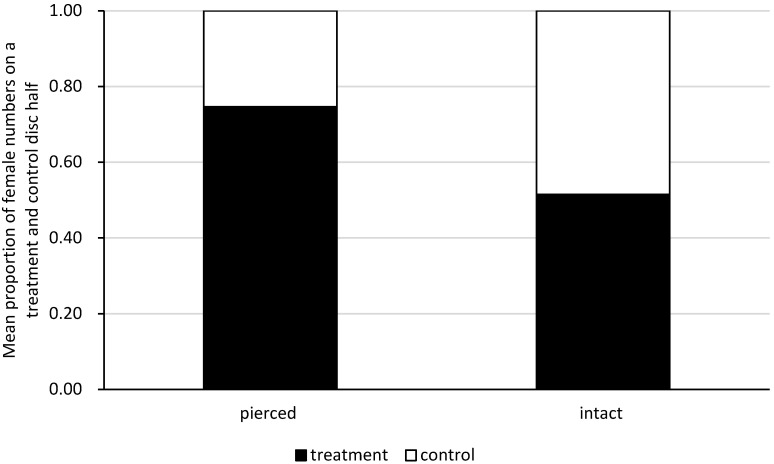
Site preference of single females on the old black locust leaf when given a choice between a leaf disc half with pierced or intact conspecifics (treatment) and the clean half of the leaf disc (control). Estimated on the basis of the pooled data on the first females’s choice and consecutive examinations of females’s presence on each disc half at one-minute intervals, throughout the subsequent 30 min from the moment of the females’s first choice.

In the choice tests, *A. allotrichus* females preferred the old black locust leaf disc half with pierced conspecifics (Wilcoxon test: V = 460.5, *n* = 19, *p* < 0.00001), but showed no preference for the leaf disc half with the intact conspecifics or the clean leaf disc half (Wilcoxon test: V = 12.43, *n* = 18, *p* = 0.3226) (Figure 3). The first choice made by a female was random, both in the experiment with the injured conspecifics (two-sided binomial test: *n* = 19, k = 15, *p* = 0.0653) and with intact conspecifics (two-sided binomial test: *n* = 18, k = 9, *p* = 1). 

## 4. Discussion

### 4.1. Attraction and Arrestment of A. allotrichus Females on Old Leaf Patches with Injured or Intact Conspecifics

In the previous study [22], both single and grouped females of *A. allotrichus* spent much more time on the old black locust leaf discs with the injured conspecifics than on the clean leaf disc. Moreover, in the detached leaf cages, grouped females put on the arena with injured conspecifics postponed their movement through the tunnel to the clean leaf patch. This suggested that the pierced conspecifics were attractive for the *A. allotrichus* females. However, the injured conspecifics might have constituted only physical obstacles that slowed down female movement and/or extended their walking patch on a leaf disc. Our present test, with the roasted, almost ‘odourless’ sand, excluded such a possibility. The sand grains were of a similar size to the eriophyoids and, applied in a similar density to the pierced individuals, did not delay female movement from the leaf discs. 

The high preference of *A. allotrichus* for the presence of injured conspecifics on the old leaves was also confirmed by the choice test. Their first choice, however, was random, which implies that the cues associated with the pierced individuals may act at a short distance only, by olfaction or taste. One cannot exclude, however, that during the tests, females became disturbed when placed on a nerve, which could have impeded their first choice and obscured the results. Thus, to determine whether the cues are attractants orientating the eriophyoids to an odour source or merely arrestants, or both, additional tests are needed. 

In contrast to *A. allotrichus*, injured conspecifics did not elicit the arrestment or attraction response of *A. guerreronis,* although their presence significantly increased the walking velocity of this eriophyoid [20]. The authors suggested that the odours of injured conspecifics may have been a clue to the presence of an eriophyoid colony, which could have modulated the exploratory behaviour in those mites. This might also be true for *A. allotrichus*. It is worth noting that under natural conditions, old black locust leaves are not inhabited by *A. allotrichus,* unless they are infested by this eriophyoid at a young developmental stage [22]. Therefore, the presence of any cue indicating the presence of the colony could arrest *A. allotrichus* females on the less profitable, old black locust leaves. The results of our tests with intact quiescent nymphs, however, only partially support this hypothesis. The presence of intact conspecifics clearly arrested *A. allotrichus* females on a leaf patch when they had to decide to move to the glass (arrestment test). However, the presence of intact individuals turned out to be ‘indifferent’ for them when they were moving from one patch to another on the same old leaf (choice test). Thus, the presence of conspecifics may be an important cue for *A. allotrichus* females when making the decision to abandon a host plant, but it appears to be insufficient when they select a leaf patch within a host plant. What could, then, be the decisive factor in the particular attractiveness of the injured conspecific for the *A. allotrichus* females? From this study, it becomes evident that it could be a mixture of various cues, not only those of eriophyoid integument, but also of the hemolymph and the digested plant sap leaking from the pierced bodies. The digested plant cell content might have been an essential signal of food for *A. allotrichus* on the non-profitable, old black locust leaves [22]. To solve this problem, however, further investigations are needed. 

To our knowledge, *A. allotrichus* is the first described herbivore in which injured conspecifics, instead of raising the alarm for foraging individuals about danger, keep them within a risky patch. Obviously, such behaviour could be destructive to *A. allotrichus* females, as it increases the chances of being preyed upon by predators. On the other hand, it could be only a by-product of our experimental set-up, as the eriophyoids were not pierced by phytoseiids but by an experimenter using a needle. Under natural conditions, the injury of conspecifics is most probably accompanied by predator cues (e.g., the predator’s saliva injected during feeding), that might in any case repel eriophyoids from such a patch. 

### 4.2. The Effect of the Presence of Injured Heterospecifics, Sand or Pollen on the A. allotrichus Movement from Old Leaves

In this study, none of the injured heterospecifics, either from the host plant or the non-host plant, elicited a threat response in *A. allotrichus* females or accelerated their movement from leaf discs. Similarly, the presence of intact heterospecifics or non-animal objects, such as cattail, rape pollen or sand grains, did not affect the time of *A. allotrichus* sojourns on leaf discs. 

As in the previous experiments, *A. allotrichus* females did not exhibit any anti-predatory behaviours in the presence of injured conspecifics [22], so we hypothesised that injured heterospecifics might not constitute a warning signal for *A. allotrichus,* either. The response to heterospecific alarm cues is expected, especially in those prey species that are closely related and/or share the same predators [26,49]. In our study, all the tested heterospecifics were phylogenetically distant to *A. allotrichus,* and only two of them, *A. craccivora* and *T. urticae,* came from the black locust. In the field, both those heterospecifics are recorded on *R. pseudoacacia* [40,50], and can potentially co-occur with *A. allotrichus* on the same black locust tree. However, while the aphid and the eriophyoid do not share the same predators (so there would be no advantage for *A. allotrichus* in utilizing cues from the damaged aphids), the two-spotted spider mites and eriophyoids do. They are both preyed upon by phytoseiid mites, which are the most dangerous enemies for both those herbivory mites [51,52]. However, the eriophyoids are only an alternative prey for the majority of phytoseiids [23,53,54]. Therefore, if anything, the injury of conspecifics, not of tetranychids, could signal the real danger for those mites. 

Contrary to our expectations, the presence of intact eggs of *T. putrescentiae* from yeast or the eggs of *T. urticae* from bean did not repel *A. allotrichus* females from the black locust leaf discs. Both these heterospecifics could be potential competitors for *A. allotrichus*, although the mould mite is omnivorous and only ‘opportunistically’ feeds on plant tissue [55,56,57]. The ability of the eriophyoid mites to recognise and respond to a competitor’s cues was demonstrated in the experiment with *A. guerreronis* [20]. The eriophyoid was exposed to the cues of tarsonemid competitors after their 24 h feeding on the plant tissue. Thus, in our test, the presence of the competitors’ eggs alone, without any cues associated with the heterospecific feeding on a leaf patch (e.g., faeces and damaged leaf tissue) might have been insufficient to repel *A. allotrichus* females. On the other hand, the cues indicating food quality and quantity may be of top priority for *A. allotrichus* females. Therefore, on non-profitable, old black locust leaf, no objects with a potentially negative effect on eriophyoid fitness could additionally enhance females’ motivation to abandon those leaves. This may include seemingly ‘neutral ‘sand or pollen grains. The investigations by [19] showed that coconut pollen did not signal the presence of a host plant and did not attract or arrest *A. guerreronis* within a patch. In our tests, the presence of non-host pollen did not affect the female sojourn on old, black locust leaves. This does not exclude, however, the possible detrimental impact of pollen or any kind of dirt, including sand grains, on eriophyoid functioning on plants. On more profitable patches, such as young leaves, the presence of competitors, as well as pollen and sand, may come to the fore, and according to their density and the degree of leaf injury, they may significantly influence the eriophyoid decision about settlement on particular plant parts. 

### 4.3. Prolonged Stay of A. allotrichus Females on Old Leaves of Non-Host Plants

When foraging or dispersing, the animals may enter a new space, and their reaction to novelty may be avoidance (neophobia) or exploratory behaviour (neofilia) [58,59,60]. Exploration enables an animal to gain information about the habitat and its profitability. However, it may also incur costs. For example, unknown food can be toxic, and unfamiliar space may contain novel predators [59]. The monophagous *A. allotrichus* can be exposed to such risks when landing on a novel plant. Therefore, to avoid possible starvation or even death, *A. allotrichus* should keep its sojourn on a non-host plant to the strict minimum and continue its dispersal quickly. In our arrestment tests, the sojourn of eriophyoid females was significantly longer on the non-host leaf discs than on those from the black locust. It must be stressed that the leaves of all tested plant species were old and sclerotised, and thus, equally ‘non-attractive’ for eriophyoid feeding. Moreover, hard kiwi vine, magnolia and maple do not possess leaf trichomes [47], which could have slowed down the movement of *A. allotrichus* females and arrested them on a leaf blade. As investigations on black bean aphid, *Aphis fabae* Scopoli, showed, dispersing individuals spent more time evaluating novel rather than familiar non-host species [61]. Similarly, the eriophyoids may need more time to recognise and evaluate the leaves of unfamiliar plants than those of the host plant. In effect, they may stay longer on the landing patch of the non-host plant than on the non-suitable feeding patch of a host plant. Phytophagous insects commonly distinguish the host from the non-host using a ratio of ubiquitous volatiles, although species-specific odours of plants can also be incorporated in this process [7,62,63,64]. So far, however, in eriophyoid, the mechanism of host plant recognition is not known. Changes in mobility parameters do not seem to describe the process of their host identification well [21]. The investigation of *A. guerreronis* suggests that gustatory or mechanical cues rather than volatiles emanating from a plant may be involved in the process of eriophyoid food source searching [18,19]. In the olfactometer experiment, the eriophyoids did not choose between plant blend and clean air, and only a small percentage of them were able to reach the bifurcation of the Y-tube [19]. However, the volatiles emitted by plants can be absorbed and desorbed on the plants’ surface [65]. Thus, one cannot exclude the possibility that apart from mechanical cues (e.g., leaf texture and pubescence) or plant taste, plant volatiles accumulated on a leaf blade or just above it, could also be used by the ‘microscopic’ eriophyoids for the recognition and evaluation of familiar and non-familiar plants. 

To summarise, this study showed that the presence of conspecifics can have a significant impact on *A. allotrichus* females on unprofitable, old black locust leaves, and can arrest them on those leaves. This effect was more pronounced in the females that were exposed to artificially injured rather than to intact individuals. They not only prolonged their stay on a leaf patch with damaged conspecifics, but also preferred the leaf patch with damaged individuals to the clean one. Similar to injured conspecifics, artificially injured heterospecifics did not raise the alarm in *A. allotrichus*, and neither did they stimulate the eriophyoid females to stay on the non-profitable leaf patches, even if they had previously fed on the black locust leaves. This may indicate the high specificity of stimulation caused by injured conspecifics. Further tests will show whether plant sap digested by conspecifics (probably indicating the presence of food) could be a specific attractant for eriophyoids on non-profitable leaf patches, and whether the *A. allotrichus* preference for leaf patches with injured conspecifics will also appear in relation to conspecifics pierced by predators. Our studies showed that neither the intact eggs of spider mites nor the eggs of mould mites repel *A. allotrichus* females from the old black locust leaves. Perhaps such leaves are simply not ‘worth competing for’, although the lack of leaf injury indicating the presence of herbivore competitors could also be the cause of the lack of the *A. allotrichus* response to the heterospecific eggs. In our tests, the presence of sand and pollen was also irrelevant to the eriophyoids, and further tests on more preferable, young leaves will show to what extent such objects can interfere with eriophyoid functioning on plants, and whether they could discourage the eriophyoids from inhabiting such leaves. Our tests using maple, hard kiwi vine and magnolia leaves revealed that *A. allotrichus* probably needs more time to identify and evaluate an unfamiliar plant than it does to identify and evaluate black locust. Do eriophyoids recognise a host plant using taxonomically specific cues or more common cues but in specific proportions? Are those cues plant volatiles or rather mechanical or gustatory infochemicals? To answer all these questions further investigations, including behavioural and electrophysiological tests, are required.

## Figures and Tables

**Table 1:** The effect of the presence of intact conspecifics, intact and injured heterospecifics, pollen and sand grains on the time spent by a single *Aculops allotrichus* female on a leaf disc. The hosts of the heterospecifics are given in brackets.

Objects on a Black Locust Leaf	Object No. per Leaf Disc	No. of Replications	Mean ± SE Time Spent by a Female on a Leaf Disc (s)		Statistics
			Treatment	Clean Black Locust Leaf (Control)	
Intact conspecifics	80	16	548.50 ± 86.23	311.69 ± 49.01	χ^2^_1,30_ = 15.379; *p* = 0.0116
Intact heterospecifics:					
mould mite (yeast)	70	15	251.4 ± 67.61	177.47 ± 47.73	χ^2^_1,28_ = 22.406; *p* = 0.3610
two-spotted spider mite (bean)	70	15	171.73 ± 25.40	135.27 ± 19.98	χ^2^_1,28_ = 11.737; *p* = 0.2538
Pierced heterospecifics:					
*Cecidophyopsis hendersoni* (yucca)	80	18	391.16 ± 81.8	270.22 ± 56.5	χ^2^_1,34_ = 31.396; *p* = 0.2122
mould mite (yeast)	70	15	192.93 ± 50.55	201.4 ± 52.77	χ^2^_1,28_ = 31.661; *p* = 0.9077
two-spotted spider mite (bean)	65	15	152.33 ± 32.08	218.13 ± 45.93	χ^2^_1,28_ = 16.558; *p* = 0.2292
two-spotted spider mite (black locust)	65	18	342.28 ± 77.45	206.56 ± 46.52	χ^2^_1,34_ = 24.948; *p* = 0.1144
cowpea aphid (black locust)	15	15	143.33 ± 43.58	172.93 ± 52.58	χ^2^_1,28_ = 16.558; *p* = 0.2292
Aggregations of rape pollen	80	15	293.00 ± 61.37	214.60 ± 44.95	χ^2^_1,28_ = 16.316; *p* = 0.2941
Aggregations of cattail pollen	80	15	271.87 ± 54.87	249.54 ± 50.37	χ^2^_1,28_ = 20.035; *p* = 0.7640
Sand grains	80	15	231.8 ± 58.65	206.6 ± 52.27	χ^2^_1,28_ = 28.408; *p* = 0.7478

**Table 2:** The effect of the presence of a non-host plant on the time spent by a single *Aculops allotrichus* female on a leaf disc.

Non-Host Plant	No. of Replications	Mean ± SE Time Spent by a Female on a Leaf Disc (s)		Statistics
		Non-Host Plant Leaf	Black Locust Leaf (Control)	
hard kiwi vine	15	462.53 ± 117.47	156.27 ± 39.69	χ^2^_1,28_ = 26.121; *p* = 0.0032
magnolia	15	454.27 ± 108.27	161.27 ± 38.43	χ^2^_1,28_ = 18.103; *p* = 0.0026
maple	15	370.2 ± 50.36	179.33 ± 24.39	χ^2^_1,28_ = 10.888; *p* = 0.0002

## Data Availability

Not applicable.
